# GlycoPP: A Webserver for Prediction of N- and O-Glycosites in Prokaryotic Protein Sequences

**DOI:** 10.1371/journal.pone.0040155

**Published:** 2012-07-09

**Authors:** Jagat S. Chauhan, Adil H. Bhat, Gajendra P. S. Raghava, Alka Rao

**Affiliations:** 1 Bioinformatics Centre, Institute of Microbial Technology, Council of Scientific and Industrial Research, Chandigarh, India; 2 Protein Science and Engineering, Institute of Microbial Technology, Council of Scientific and Industrial Research, Chandigarh, India; King’s College London, United Kingdom

## Abstract

Glycosylation is one of the most abundant post-translational modifications (PTMs) required for various structure/function modulations of proteins in a living cell. Although elucidated recently in prokaryotes, this type of PTM is present across all three domains of life. In prokaryotes, two types of protein glycan linkages are more widespread namely, N- linked, where a glycan moiety is attached to the amide group of Asn, and O- linked, where a glycan moiety is attached to the hydroxyl group of Ser/Thr/Tyr. For their biologically ubiquitous nature, significance, and technology applications, the study of prokaryotic glycoproteins is a fast emerging area of research. Here we describe new Support Vector Machine (SVM) based algorithms (models) developed for predicting glycosylated-residues (glycosites) with high accuracy in prokaryotic protein sequences. The models are based on binary profile of patterns, composition profile of patterns, and position-specific scoring matrix profile of patterns as training features. The study employ an extensive dataset of 107 N-linked and 116 O-linked glycosites extracted from 59 experimentally characterized glycoproteins of prokaryotes. This dataset includes validated N-glycosites from phyla *Crenarchaeota*, *Euryarchaeota* (domain Archaea), *Proteobacteria* (domain Bacteria) and validated O-glycosites from phyla *Actinobacteria*, *Bacteroidetes*, *Firmicutes* and *Proteobacteria* (domain Bacteria). In view of the current understanding that glycosylation occurs on folded proteins in bacteria, hybrid models have been developed using information on predicted secondary structures and accessible surface area in various combinations with training features. Using these models, N-glycosites and O-glycosites could be predicted with an accuracy of 82.71% (MCC 0.65) and 73.71% (MCC 0.48), respectively. An evaluation of the best performing models with 28 independent prokaryotic glycoproteins confirms the suitability of these models in predicting N- and O-glycosites in potential glycoproteins from aforementioned organisms, with reasonably high confidence. A web server GlycoPP, implementing these models is available freely at http:/www.imtech.res.in/raghava/glycopp/.

## Introduction

Glycosylation is a recently identified post-translational modification of proteins in prokaryotes: Archaea and Bacteria [1], [2]. A glycan moiety is attached enzymatically to a protein by the process of glycosylation. Glycosylation is known to influence biological properties like activity, solubility, folding, conformation, stability, half-life, and/or immunogenicity of different cellular proteins thereby modulating the structure/function of these proteins for a variety of cellular/extracellular functions in a living cell [3]–[5]. Owing to their involvement in host-pathogen interactions, immunogenicity and in many other important cellular functions, a number of bacterial and archaeal glycoproteins have been characterized experimentally [6]–[10]. Determination of glycosite(s) is one important aspect of glycoprotein characterization. Analysis of glysosites and their neighboring sequence and structural contexts may also provide important evolutionary insights and understanding of acceptor specificities of the protein glycosylating enzymes called glycosyltransferases (GTs) and oligosaccharyltransferases (OSTs), [2]. The experimental characterization of glycosite(s) and the glycoproteins, however, could be difficult, technically demanding, and time-consuming owing to the labile nature of modification involved as well as lack of high-senstivity yet cost-effective methods for glycoprotein detection. Therefore, the computational algorithms/models to predict glycosites in protein sequences are very useful in complementing and facilitating such studies. A number of such algorithms have been developed to predict glycosites in eukaryotic glycoproteins using different tools of machine learning like Neural Network based (NetOglyc), [11], [12] Support Vector Machine (SVM) based (NetNglyc), [13], Ensemble of SVMs (EnsembleGly), [14] and Random Forest based [15]. All these existing tools are trained on eukaryotic glycoprotein sequences. However, for non-availability of equivalent methods, these tools are routinely used for analyzing glycoproteomics data and potential glycosite analysis in prokaryotic glycoproteins for both N- and O- type of glycosylation [16]–[19]. In similar context, Dell and co-workers have discussed the unsuitability of these existing glycosite prediction tools in correctly predicting glycosites (especially O-glycosites), in most families of characterized prokaryotic glycoproteins that included pilins, flagellins, autotransporters and serine-rich proteins [20]. In this study using a dataset of experimentally validated 107 N-linked and 116 O-linked glycosites from archaeal and bacterial glycoproteins, we have found that indeed these tools (as detailed in Table 1), fail to provide reliable predictions for glycosites in prokaryotic glycoproteins. Furthermore, protein glycosylation in prokaryotes is much more versatile than in eukaryotes in terms of both mechanisms involved and the types of glycans and linkages present as discussed in references [20]–[22] & Table 2. Among archaea N-glycosylation is believed to be widespread yet experimental evidence exists only in case of phyla *Crenarchaeota* and *Euryarchaeota* where it is mediated by an enzyme AglB and its homologues and sugar is transferred on to NX(S/T)(where X≠P) acceptor sequon in an *en-bloc* fashion. Similarly, in bacteria N-glycosylation is known and experimentally validated only in a few organisms belonging to phylum *Proteobacteria*. In *Proteobacteria* both sequentially (in cytoplasm, ex. *Haemophilus influenzae*) and *en-bloc* glycosylated (in periplasm, ex. *Campylobacter jejuni*) proteins have been characterized in different organisms. Similarly, experimental data on O-glycosites is available only from four bacterial phyla namely, *Actinobacteria*, *Bacteroidetes*, *Firmicutes* and *Proteobacteria* out of eleven bacterial phyla where glycoproteins are known to exist. Interesting novel “conserved sequences of amino acids around glycosites (sequons)” like (D/E)X_1_NX(S/T)(where X_1_ & X≠P) for N-glycosylation and D(S/T)(A/I/L/V/M/T) for O-glycosylation have been elucidated within these glycoproteins that are not yet seen in eukaryotes [23], [24]. A tool to map such sequons in amino acid sequence(s) of protein/proteomes has recently been made available by our group [6]. Further, an analysis of amino acid sequences surrounding N-glycosites of available archaeal glycoproteins (10 at that time) by Abu-Qarn and co-workers has also shown that archaeal N-glycosites are rarely surrounded by aromatic residues that are in abundance at positions –2 and –1 preceding glycosylated Asn at postion 0 in eukaryotic N-glycosites [25]–[27]. For these reasons, the development of separate and new algorithms for prediction of glycosites in prokaryotes is of high interest and need [20].

**Table 1:** An evaluation of performances of some of the well-known models for glycosylation prediction on prokaryotic glycoproteins.

Type of Glycosylation	Prediction Tools	Threshold	Sensitivity (%)	Specificity (%)	Accuracy (%)	MCC (%)
N-linked	NetNglyc^1^	0.5	81.75	10.16	34.41	−0.11
		0.6	50.79	42.68	45.43	−0.06
		0.7	15.87	76.02	55.65	−0.09
		0.9	0.79	98.37	65.32	−0.03
	EnsembleGly^3^	0.3	92.86	0.41	31.72	−0.2
		0.5	90.48	1.63	31.72	−0.18
		0.7	79.37	10.98	34.14	−0.13
		0.9	51.59	47.15	48.66	−0.01
O-linked	NetOglyc^2^	0	8.38	95.64	87.96	0.05
	EnsembleGly^3^	0	9.5	93.37	86	0.03

Footnotes: (1: http://www.cbs.dtu.dk/services/NetNGlyc/, 2: http://www.cbs.dtu.dk/services/NetOGlyc-3.0/, 3: http://turing.cs.iastate.edu/EnsembleGly/).

**Table 2:** Experimentally characterized glycan linkages at known glycosites of bacteria and archaea.

Sugar linkage	Class	Example glycoproteins
**N-LINKED GLYCANS/ARCHAEA**		
Glc-Asn	*Halobacteria*	Flagellin, Slg
βGalNAc- Asn,	*Halobacteria, Methanococci, Methanobacteria Thermoprotei*	Flagellin, Slg, Cytochrome subunit
**N-LINKED GLYCANS/BACTERIA**		
Bac-Asn	*Epsilonproteobacteria*	AcrA, PEB3, CgpA, HisJ, ZnuA, jlpA etc.
GlcNAc-Asn	*Deltaproteobacteria*	HmcA
Hexose-Asn, dihexose-Asn, Glu-Asn,Gal-Asn	*Gammaproteobacteria*	Adhesins
**O-LINKED GLYCANS/BACTERIA**		
Man-Ser/Thr	*Actinobacteria, Flavobacteria, Sphingobacteria*	Glycosidases, Cell surface lipoproteins,Secreted antigens, Superoxide dismutase, Heparinase, Chondroitinase etc.
Fucose	*Bacteroidia*	Putative cell division proteins, exported proteins, outer membrane proteins etc.
β-GalNAc-Ser/Thr	*Bacilli*	Slg
β-D-Gal-Ser/Thr	*Bacilli*	Slg, SgsE, SgtA etc.
β-GlcNAc-Ser/Thr, HexNAc	*Bacilli*	Glycocin F, Flagellin
Bac/DATDH-Ser	*Betaproteobacteria*	Pilin, CcoP, CycB etc.
FucNAc-Ser	*Gammaproteobacteria*	Pilin
Rha-Ser/Thr, Deoxyhexose-Ser	*Gammaproteobacteria*	Flagellin

Footnotes: Detailed information about attached glycan and glycoproteins can be obtained from www.proglycprot.org).

**Figure 1:**
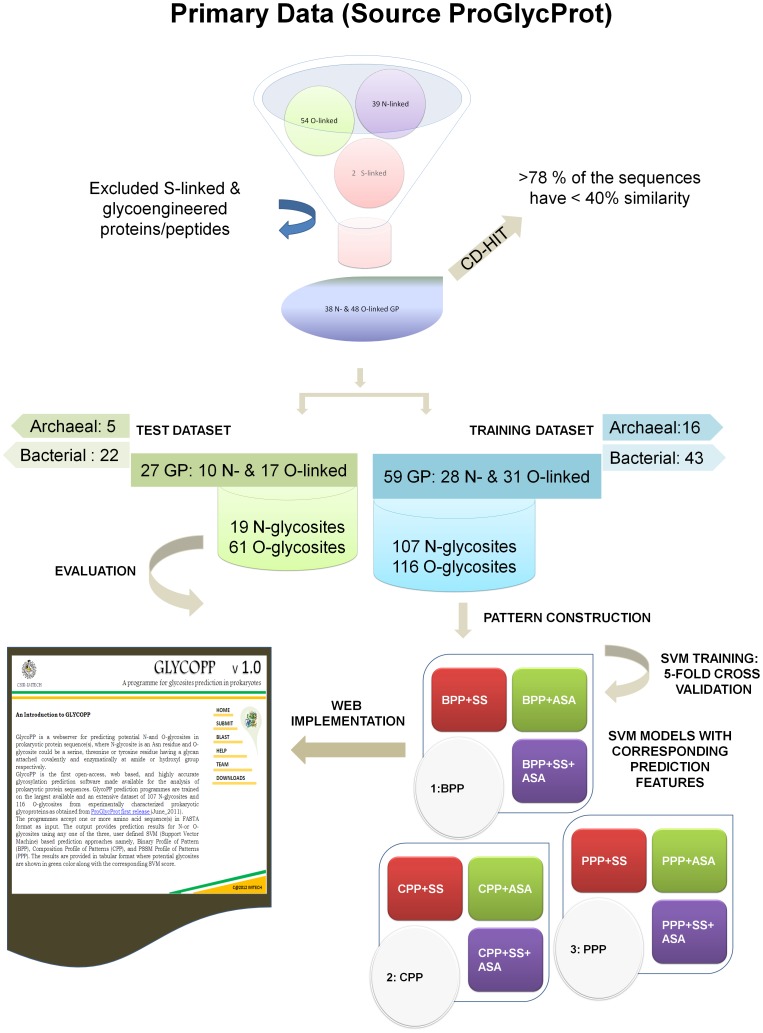
GlycoPP websever Schema. A flowchart of methodologies employed for development of GlycoPP webserver for prediction of N & O-glycosites in prokaryotic protein sequences.

Therefore in this study, we have attempted to analyze the sequence context, predicted secondary structure and surface accessibility of the experimentally verified glycosites in the largest available dataset of 107 N-linked glycosylated-residues (N-glycosites) and 116 O-linked glycosylated-residues (O-glycosites) from 59 prokaryotic glycoproteins retrieved from our recently published database of experimentally characterized prokaryotic glycoproteins, ProGlycProt [6]. In this study, we have developed a number of SVM models using three types of features namely, binary profile of patterns (BPP), composition profile of patterns (CPP), and PSI-BLAST generated PSSM profile of patterns (PPP) to recognize and differentiate glycosylated sequence contexts from non-glycosylated contexts in prokaryotic glycoproteins. For the reasons that mere presence of a consensus-sequon/pattern may not always be sufficient for glycosylation to occur and that the glycosites are predominantly situated on loops/accessible portions of folded proteins in prokaryotes, we have employed predicted secondary structure and surface accessibility features in combination with BPP, CPP and PPP for developing hybrid models (Table 3), [21], [28]. The best performing and significantly accurate models were then evaluated against an independent dataset of experimentally validated glycosites and finally implemented via web server GlycoPP (Figure 1) made available through open access at http:/www.imtech.res.in/raghava/glycopp/.

**Table 3:** An analysis of experimentally observed secondary structures in prokaryotic glycosites.

Protein Name (Source organism)	PDB ID	Presence of glycanin structure	Validated Glycositesin full length protein sequence	Position of Glycositesin PDB entry sequence	SS
**N-Glycosylated Proteins**					
Tetrabrachion(Staphylothermus marinus)	1YBK,1FE6	–	N44, N605, N641,N685, N708, N1279,N1402	N44 (N1279*)	**H^1^**
Chondroitinase ABC(Proteus vulgaris)	1HN0	–	N282, N338, N345,N515, N675, N856,N963	N282, N963 & N675N338, N345 & N515N856	**FR^1^ H^1^ B^1^**
PotD (Escherichia coli)	1POT,1POY	–	N26, N62	N26N62	**FR^3^ FR^1^ (at beginning of helix)**
AcrA (Campylobacter jejuni)	2K32,2K33(NMR)	Heptasaccharide	N123, N273	N42 (N123*)	**FR^1^**
PEB3 (Campylobacter jejuni)	2HXW	-	N90	N90	**FR^1^ (between helices)**
HmcA (Desulfovibrio gigas)	1Z1N	Trisaccharide (NAG,NAA,any epimer of NAG),	N290	N261	**FR^2^ (between beta-sheets)**
**O-Glycosylated Proteins**					
Chondroitinase-AC(Pedobacter heparinus)	1CB8,1HM2,1HM3,1HMU,1HMW	Tetrasaccharide Man-(Rha)-GlcUA-Xyl,	S328, S455	S328 S455	**FR^1^ (just after helix)** **FR^1^ (between beta-sheets)**
Chondroitinase-B(Pedobacter heparinus)	1DBG,1DBO,1OFL,1OFM	Heptasaccharide galactose-β(1–4)[galactose-α(1–3)](2-O-Me)fucose-β(1–4)xylose-β (1–4)glucuronicacid-α(1–2)[rhamnose-α(1–4)]mannose-α(1-	S234	S234	**FR^1^ (between beta-strands)**
Heparinase II(Pedobacter heparinus)	2FUQ,2FUT	Tetrasaccharide Man-(Rha)-GlcUA-Xyl (xylose-β(1–4)glucuronic acid-α(1–2)[rhamnose-α(1–4)]mannose-α(1-	T134	T134	**H^3^**
Fimbrial protein(Neisseria gonorrhoeae)	2HI2,2HIL,2PIL,1AY2	Disaccharides α-D-galactopyranosyl-(1→3)-2,4-diacetamido-2,4-dideoxy-β-D-glucopyranoside(bacillosamine, Bac);Gal-DADDGlc; andGlcNAc-α1,3-Gal	S70	S63	**H^1^ (before helix)**
Glycocin F(Lactobacillus plantarum)	2KUY(NMR)	Two N-Acetylglucosamines	S39	S18	**FR^3^**
Endo-β-N-acetylglucosaminidase F3 (Flavobacterium meningosepticum)	1EOM,1EOK	-	T88	T49	**FR^1^**

Footnotes: All crystal structures are obtained from www.rcsb.org. All structures are at a resolution of 1.4 Å or above.

Symbols used: - : No sugar detected, *: Corresponding position in full length protein sequence, F: flexible Regions with turns/loops/coils/bends or no assigned secondary structure, H: helix, B: beta sheet, 1: Intra domain, 2: Interdomain, 3: no assigned domain.

## Methods

### Dataset Generation

#### Source of data

The primary set consisted of 39 N-linked and 54 O-linked glycoproteins obtained from the first release (July_2011) of ProGlycProt database [6]. For the reason that number of experimentally validated proteins is not very high, all the available N-linked and O-linked glycoprotein entries in primary dataset have been taken in to account for this study. However, entries containing only cysteine-linked (S-linked) glycosites as well as all glyco-engineered protein/peptides have been excluded from the primary set resulting into a total of 38 N-linked and 48 O-linked glycoproteins for further consideration. Some of these glycoproteins are N- as well as O-glycosylated. These glycoproteins include a variety of important proteins like S-layer proteins, flagellar proteins, pili/fimbrial proteins, lectins, adhesions, glycosidases, Cytochrome hemoprotein, heparinase, Chondroitinase as well as several known-unknown cytoplasmic, membrane bound and exported proteins (Table 2). These glycoproteins represent all types of known N-glycosylation in prokaryotes representing organisms from phylum *Crenarchaeota* and *Euryarchaeota* of Archaea and phylum *Proteobacteria* of Bacteria. Similarly, this dataset represents all available validated examples of O-glycosylated proteins from four phyla namely, *Actinobacteria*, *Bacteroidetes*, *Firmicutes* and *Proteobacteria* of Bacteria. In Archaea no experimentally validated data exists for O-glycosites, so far. Further, within these glycoproteins, at least 30 N-linked and 40 O-linked glycoproteins have less than 40% sequence similarity to each other as deduced from CD-HIT v 4.0 available at http://www.bioinformatics.org/cd-hit/. From the primary dataset, 59 glycoproteins (16 archaeal and 43 bacterial) with higher number of characterized glycosites were hand- picked to form main datasets whereas remaining 27 (5 archaeal and 22 bacterial) glycoproteins were used as independent datasets of N and O glycosites, separately.

#### Main datasets

The Main datasets represent the training datasets employed in profile generation and later machine learning. The datasets contain 28 N-linked (overall sequence similarity less than 70%) and 31 O-linked (overall sequence similarity less than 90%) glycoproteins from prokaryotes. Using CD-HIT it has been deduced that in the main datasets, at least 23 N-linked and 26 O-linked glycoproteins have less than 40% sequence similarity to each other. From this set of glycoproteins, all N-and O-glycosites were retrieved and segregated in to separate datasets. All probable or predicted glycosites were excluded. Finally, the main datasets contained well-annotated unambiguous 107 N-linked and 116 O-linked glycosites derived from 59 experimentally validated prokaryotic glycoproteins. The O-linked glycosites (116) exclusively consisted of bacterial glycosites for unavailability of experimentally validated archaeal O-glycosite(s) [6]. To our knowledge, these are the **most extensive** datasets of experimentally validated prokaryotic glycosites (and glycoproteins), employed to develop **first** glycosite prediction models trained on and for prokaryotic protein sequences. These datasets are further divided in to two subgroups as follows.

**Balanced datasets** derived from randomly selecting all positive instances (positive training datasets) and equal number of negative instances (negative training datasets) across the protein lengths. Balanced datasets are useful in accelerating the machine learning and in avoiding biases in machine learning that are common in case of realistic dataset.

**Realistic datasets** contained all glycosylated/positive (107 N-linked & 116 O-linked) and all non-glycosylated/negative (995 N-linked & 2018 O-linked) sites from glycoprotein sequences. Performance of SVM on realistic (unbalanced) datasets could provide more confidence in predictions from real-time data where usually the non-glycosylated residues are much more than the glycosylated ones in a protein sequence.

#### Independent datasets

The independent balanced datasets of 28 (10 N-linked & 17 O-linked) glycoproteins with experimentally validated 19 N-glycosites and 61 O-glycosites (with equivalent numbers of non-glycosylated sites) were used as test datasets in this study for evaluating the models trained on main datasets. Within these at least 7 N-linked and 14 O-linked glycoproteins show less than 40% sequence similarity to each other.

### Pattern Generation and Feature Calculations

Various overlapping symmetrical sequence patterns of residues length 21 that included central glycosylated residues were constructed according to previous studies [14], [15]. A sequence pattern was considered positive if central residue was glycosylated otherwise the same was assigned as a negative pattern. To generate a pattern corresponding to the terminal residues in a protein sequence of length L, dummy residues “X” in number (L-1)/2 were added at both the termini of the protein [29], [30].

#### Binary profile of patterns (BPP)

Fixed length of 21 residues in sequence patterns was converted into binary form according to the existing study [30]. Each residue of patterns was represented by a vector of dimension 21 (e.g. Ala by 1,0,0,0,0,0,0,0,0,0,0,0,0,0,0,0,0,0,0,0,0; Cys by 0,1,0,0,0,0,0,0,0,0,0,0,0,0,0,0,0,0,0,0,0), which contained 20 amino acids and one dummy amino acid “X”.

#### Composition profile of patterns (CPP)

Composition profile of patterns is the percentage frequencies of each amino acid in a fixed length sequence pattern. The fractions of all 20 natural amino acids of fixed length sequence patterns were calculated using the following equation [30]:
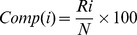
Where *Comp(i)* is the percent composition of amino acid residue of type *i*; *Ri* is number of amino acid residues of type *i*, and *N* is the total number of residues in the fixed length sequence pattern.

#### PSSM profile of patterns (PPP)

In addition to compositional information, PSSM provides important information of evolutionary significance about residue conservation at a given position in a protein sequence. The multiple sequence alignment information in the form of position specific scoring matrix (PSSM) has been used here to develop learning model where each glycosylated protein sequence was first searched against ‘SWISS-PROT’ database followed by generation of alignment profiles or position specific scoring matrices (PSSM) using PSI-BLAST v 2.2.20 program (ftp://ftp.ncbi.nlm.nih.gov/blast/executables/blast/LATEST/). Three iterations of PSI-BLAST were run for each protein with cut off e-value 0.001. We have normalized each value range between 0 to 1 using sigmoid function by following equation, where *val* is the PSSM score and *Val* is its normalized value [29]–[31]:
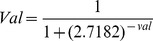



#### Secondary structure information

For this study, the secondary structure (SS) information (coil/helix/sheets) for glycosylated residue and its sequence context was obtained using webserver PSIPRED v 3.21 available at http://bioinfadmin.cs.ucl.ac.uk/downloads/psipred/ [32].

#### Surface accessibility information

The accessible surface area (ASA) is the surface area of a protein that is accessible to another protein or ligand(s). For our analysis, the average accessible surface area values of each amino acid were predicted from Sarpred available at www.imtech.res.in/raghava/sarpred/ [33].

### Support Vector Machine (SVM) Algorithm and Evaluation Models

The SVM is a supervised machine-learning technique based on the structural risk minimization principle [34]. In this study, we have used freely available SVM^light^ classifier v 6.01 (http://svmlight.joachims.org/) where we could adjust the parameters and kernel (linear, polynomial, radial basis function, sigmoid) functions. The advantage of SVM over other machine learning techniques is that it can be trained on small dataset (as in this study) with minimum over-optimization. SVM based approach has been successfully employed in developing both N- and O-glycosylation prediction tools for mammalian glycoproteins in past [13], [14]. Using different sequence properties like identity and position of residues (BPP), percentage composition of residues (CPP), residue conservation information (PSSM) along with structural features like secondary structure and surface accessibility several SVM classifiers have been trained and optimized for this study. Our group has successfully used one or more of these features in predicting GTP interacting residues, Mannose interacting residues, in predicting Cyclin protein sequences and in identification of conformational B-cell Epitopes from primary sequences of proteins, previously [29], [30], [35], [36]. In this study, a 5-fold cross-validation procedure has been used to develop the prediction model, where five subsets were constructed randomly from the main datasets. At a given point of time, the models were trained on four sets of the training dataset and the performance was measured on the remaining fifth set. This process is repeated five times in such a way that each set was used once for testing. The final performance was obtained by averaging the performances of all five sets. The models thus obtained were evaluated for performance using threshold dependent parameters namely, sensitivity (Sn), Specificity (Sp), Accuracy (Acc), Matthews correlation coefficient (MCC) as well as using threshold dependent parameters Area Under Curve (AUC) values.

Evaluation parameters employed in this study are described briefly as below:

**Sensitivity** is the percentage of glycosites that are correctly predicted as glycosylated:
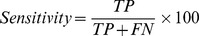



**Specificity** is the percentage of non-glycosylated sites that are correctly predicted as non-glycosylated:
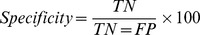



**Accuracy** is the percentage of correct prediction out of total number of predictions:
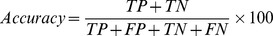



**Matthews correlation coefficient** (MCC) is a measure of both sensitivity and specificity. MCC value would range from 0 (indicating completely random prediction) to 1 (indicating perfect prediction):
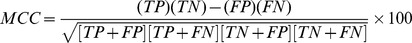
[Where TP- true positive; FN- false negative; TN- true negative; FP- false positive]

Threshold selection is important criteria for checking the consistency of prediction results. In our study, we have varied threshold in the range of –1 to +1, normally we selected “0” as default threshold to achieve balance between sensitivity and specificity.

Area Under Curve (AUC) a threshold independent parameter describes inherent trade-off between sensitivity and specificity. Receiver Operating Characteristic (ROC) plots were drawn between TP rate (sensitivity) and FP rate (1-specificity) using R-package v 2.14.1 (http://www.r-project.org/) to calculate AUC values. Finally, the best performing models in terms of accuracy & MCC values were validated using an independent dataset of prokaryotic glycoproteins for final implementation at GlycoPP webserver (Figure 1).

## Results

### Prediction Performance of Some of the Existing Tools on Prokaryotic Glycoproteins

In order to evaluate the suitability of models trained on eukaryotic glycoproteins for predicting glycosites in prokaryotic proteins, the proteins of main datasets were run on three of the well-known prediction tools for prediction of N- and O-glycosites. Against the experimentally validated glycoproteins of prokaryotes, the performances of these tools were found very poor and are detailed in Table 1. From this, we conclude that the methods that are trained using eukaryotic glycoprotein are not optimum for prediction of potential glycosites in bacterial and archaeal proteins. This also suggests that the sequence or structural contexts around prokaryotic glycosites could be different from what is known in eukaryotic glycosites. This is logical as several OSTs with novel mechanisms of sugar transfer on to the acceptor proteins are now known in bacteria as well as archaea. This prompted us to develop a number of new algorithms to recognize and differentiate glycosylated and unglycosylated sequence contexts of known glycosites of archaeal and bacterial proteins representing aforementioned six different phyla. These algorithms are trained using different input features and described in this study.

### Sequence Context of Prokaryotic Glycosites

**Figure 2:**
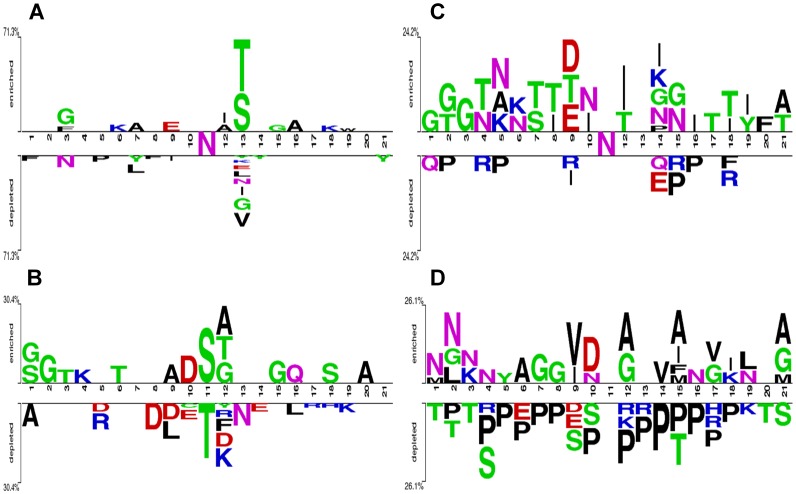
Sequence contexts of prokaryotic glycosites. Two sample weblogos depicting enriched and depleted amino acids around prokaryotic N-glycosites (logo A) and prokaryotic O-glycosites (logo B) in comparison to the percentage of these amino acids around non-glycosylated prokaryotic N-glycosites and O-glycosites, respectively. Similarly, logos C and D provide an assessment of probabilities of amino acids around prokaryotic N- and O-glycosites in comparison to probabilities around eukaryotic N- and O-glycosites, respectively. The datasets for eukaryotic N- and O- glycosites for generation of weblogos is obtained from SWISS-PROT (2011 release).

In an attempt to understand the general preferences for different amino acids around prokaryotic glycosites as well as the differences from the corresponding sequences in eukaryotes, we have generated a number of one sample and two sample weblogos (http://weblogo.berkeley.edu/& http://www.twosamplelogo.org/) for N- and O-glycosites of archaeal and bacterial glycoproteins in an organism specific, phylum specifc as well as domain specific manner, respectively. The interesting existing knowledge as well as our statistically significant observations for the purposes of a prediction model are discussed here, briefly. Similar to eukaryotic glycoproteins, the minimal sequon NX(S/T)(where X≠P) is essential for N-glycosylation in prokaryotic glycoproteins. For example in all archaeal glycoproteins (Figure S1), [25], in HmcA protein of *Desulfovibrio* [37], adhesin protein HMW1 of *Haemophilus influenzae* and *Actinobacillus pleuropneumoniae* (where glycosylation is sequential and mediated by a novel cytoplasmic glycosyltransferase, HMW1C of family GT41, Figure S2), [38]. However, as known already, the sequon is extended as (D/E)X_1_NX(S/T)(where X_1_ & X≠P) but not stringent in case of PglB (OST of *Campylobacter*) mediated *en bloc* N-glycosylation in *Campylobacter* and *Helicobacter* (Figure S2), [39]. As discussed before, the first-ever defined sequon D(S/T)(A/I/L/V/M/T) for O-glycosites is indeed conserved across available glycoproteins from three representative classes including *Bacteroidia*, *Flavobacteria* and *Sphingobacteria* of phylum *Bacteroidetes* (Figure S3). Further, the two-sample logos comparing prokaryotic and eukaryotic N-and O- glycosites clearly illustrate the differences in the amino acid preferences around these glycosites (Figure 2), indicating a necessity for independent prediction tool for prokaryotes. With respect to glycosylated Asn (if at position 0) the positions at -1 and -2 have previously been stated to be enriched in aromatic amino acids in eukaryotic N-glycosites [25]–[27]. However, in prokaryotic N-glycosites instead we observe a marked preference for polar residues like Asp/Glu/Thr/Asn and lysine at different positions preceding glycosylated Asn (Logo C, Figure 2). Similarly, at positions -2 and -6 occurrence of polar residues is higher around NX(S/T) motif in validated N-glycosites of prokaryotes in contrast to randomly selected equal number of NX(S/T) motifs with unglycosylated Asn from prokaryotic glycoproteins (Logo A, Figure 2). An analysis of eukaryotic N-glycosites by Pertescu and co-workers had suggested a preference for small hydrophobic residue at positions +1 and large hydrophobic residue at +3 in eukaryotes, previously [27]. Similarly, in case of prokaryotic glycoproteins hydrophobic residues are though present at +1 position yet preference for large or small residues are not very clear (Figure 2, Figure S1), [25]. Furthermore, increased instances of Pro near the glycosylated residues are not observed in bacterial and archaeal glycoproteins as found in eukaryotic glycoproteins. Instead Pro is one of the significantly depleted amino acids at +4 and +5 positions here [27]. Likewise, sequence surrounding all prokaryotic O-glycosites (Figure 2) is different in having higher instances of Gly, Ala, Val and a significant depletion of Pro at almost all positions (except in mannosylated glycoproteins of *Mycobacterium spp,* Figure S2), [8] in comparison to the eukaryotic mucin type O-glycosites that are rich in Ser, Ala and Pro (Logo D, Figure 2), [12]. In prokaryotic O-glycosites, apart from this general presence of small hydrophobic amino acids around 10 residues on either sides of glycosylated Ser/Thr residues (at position 0), a marked preference for negatively charged Asp at -1 that in fact is a part of potential sequon for O-glycosites in *Bacteroidetes* is observed (Logo B, Figure 2).

### Structural Features of Prokaryotic Glycosites

Previous statistical analysis of all available crystal structures of eukaryotic glycoproteins by Petrescu et al. had suggested that the probability of finding N-glycosites was higher at positions where there was a secondary structure change [27]. Upon analysis of 12 eukaryotic glycoproteins, Julenius et al had also concluded that O-glycosites mainly occurred in coil region of mucin type of O-glycosylated proteins [12]. The secondary structure and surface accessibility of a residue therefore are considered important criteria in prediction of glycosites in eukaryotes. Some of the existing eukaryotic glycosite prediction models have employed these features successfully [11], [12]. Unlike eukaryotic N-glycosylation that is a co-translational event, the glycosylation is considered a true post-translational modification in bacteria where the folding state of a polypeptide/protein could dictate availability of a sequon/site for glycan attachment on to a protein [21]. Although limited, yet most of the X-ray crystal structures and NMR structures of bacterial glycoproteins (as listed at http://www.proglycprot.org/CrystalStructure.aspx) show that the glycosylated residues are indeed primarily located in surface-exposed flexible loops/turns/bends that then should be accessible to bacterial OSTs/GTs. The structural contexts for 20 glycosites (13 N- & 7 O-glycosites) extracted from available structures of N- and O-glycoproteins of Archaea and Bacteria, reveal that at least 65% (13 out of 20) of these glycosites are located in aforementioned flexible regions and primarily in intra-domain region (Table 3). Incidentally, at least three of the N-glycosylated proteins namely, PotD of Escherichia coli, AcrA and PEB3 of *Campylobacter jejuni* are glycosylated (*in vitro/in vivo*) by OST of *Campylobacter* (PglB) that has previously been shown to transfer sugars post-translationally to locally flexible structures in folded proteins [21], [28], [40]. Similarly, glycosylated Ser/Thr residues in Endo-β-N-acetylglucosaminidase F3 (*Flavobacterium meningosepticum*), Chondroitinase-AC and Chondroitinase-B (*Pedobacter heparinus*) lie in the similar loops/bends in their respective crystal structures (Table 3).

**Figure 3:**
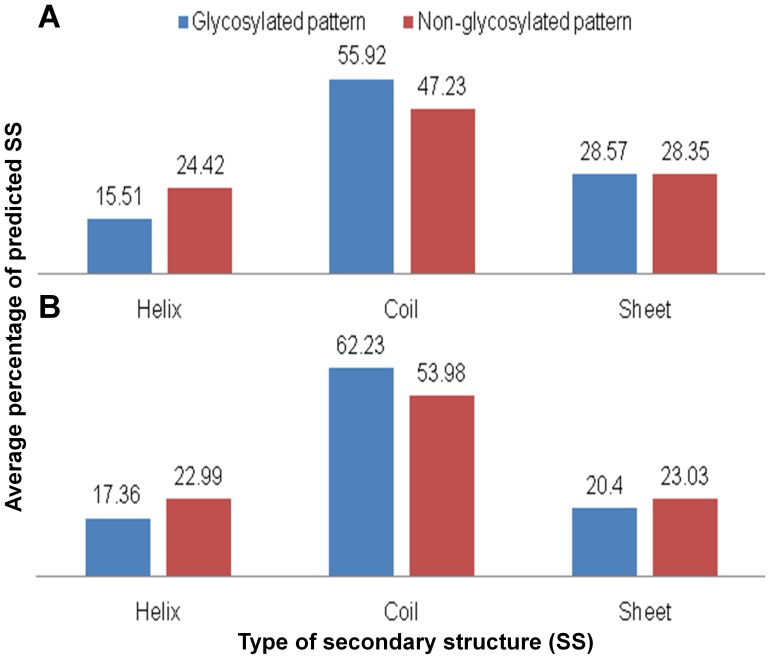
Predicted secondary structures around prokaryotic glycosites. Average percentage of secondary structures predicted in and around N-glycosites (panel A) and O-glycosites (panel B) in prokaryotic glycoproteins. The graph indicates a general likelihood of locating a glycosylated residue in coils/turns in a protein.

**Figure 4:**
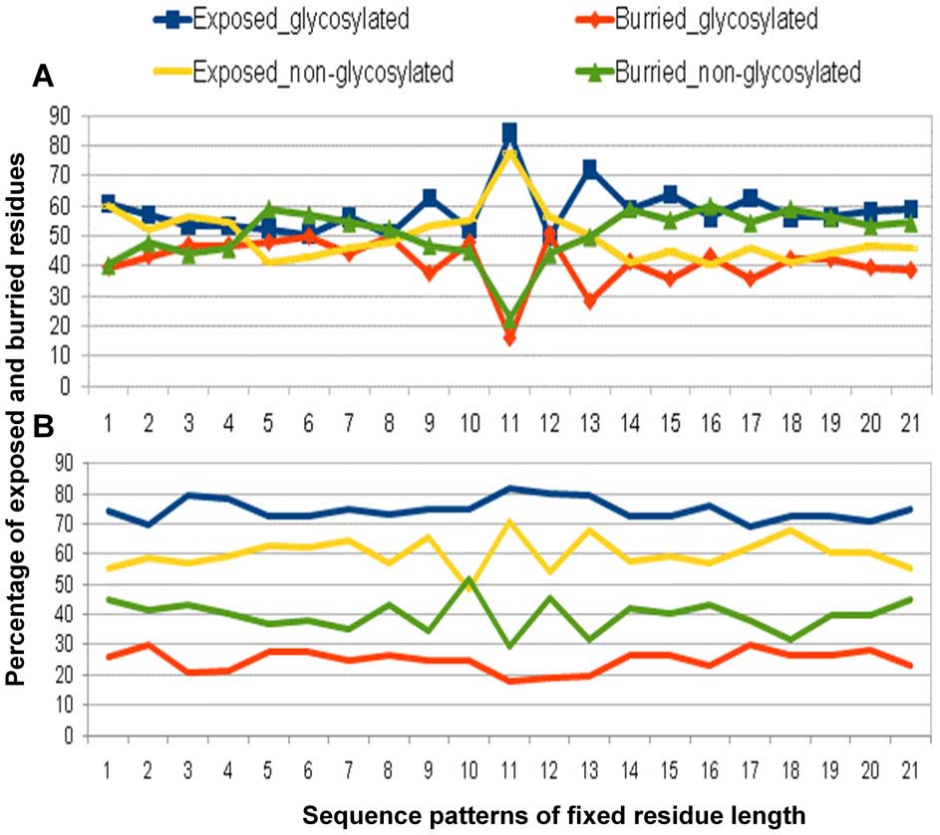
Predicted Surface accessibility of prokaryotic glycosites. Average percentage of exposed and buried residues predicted in and around N-glycosites (panel A) and O-glycosites (panel B) in prokaryotic glycoproteins. The graph suggests higher accessibility of glycosylated residues on surface of a protein in comparison to non-glycosylated ones.

Our analysis of the predicted secondary structure indicates that 55.92% of the validated glycosites are found in coils, 15.51% in helix and 28.57% in sheets whereas non-validated glycosites or their sequence contexts are found correspondingly less in coil (47.23%), more in helix (24.42%) and almost equally in sheets (28.35%). Similarly, 17.36% of validated O-glycosites are situated in helix, 62.63% in coils and and 20.4% in sheets in contrast to non-validated O-glycosites that are found more often in helix (22.99%) and less in coils (53.98) and almost equally in sheets (23.03), respectively (Figure 3). Similarly, predicted surface accessibility profile of glycosylated Asn residues suggest them to be much more surface accessible than the corresponding non-glycosylated sequence contexts as shown in Figure 4. The glycosylated Ser/Thr are again, more accessible (80%) compared to the non-glycosylated residues (60%). To summarize, most of the prokaryotic glycosites (both N as well as O) indeed seems to be present in flexible and exposed regions. Further, not only the central glycosylated-residues but also their surrounding residues are highly accessible and surface exposed.

### Prediction Performance of SVM Using Balanced Datasets

SVM models based on BPP, CPP and PSSM profiles are well recognized for their notable performances in predicting a variety of motifs and interactions in biomolecules and have been used effectively in the past for glycosites predictions as well [29]–[31], [35], [36]. Accordingly, we have generated several SVM models using BPP, CPP and PPP profiles as input features. The performance measures were calculated at different thresholds of SVM scores ranging from −1.0 to 1.0 and the best performing thresholds were selected for further optimization. The prediction of the N- glycosites were best achieved by the SVM models developed using BPP profile achieving 79.91% accuracy and 0.60 MCC (Table 4) whereas O-glycosites were best predicted by SVM model developed using PPP with 74.57% accuracy and 0.49 MCC (Table 5). As discussed before, sequence features namely secondary structure (SS) and accessible surface area (ASA) could play an important role in correct predictions of sites of glycoslation in a protein. Therefore, we have developed prediction models with these features in following three combinations: (i) composition profile of patterns with either secondary structure or surface accessibility or both (ii) Binary profile of patterns with either secondary structure or surface accessibility or both (iii) PPP with either secondary structure or surface accessibility or both. In general, inclusion of SS and SAS profiles in prediction models helped improvise predictions (Table 4, Table 5, Figure S4). The hybrid model of BPP+ASA proved as good as BPP+SS+ASA improving the maximum MCC of prediction from 0.60 to 0.65 and accuracy of prediction from 79.91% to 82.24% for N-glycosites (Table 4). Similarly, predictions of O-glycosites were improvised slightly using the hybrid model (based on combination of PPP+SS+ASA profiles) giving MCC of 0.48 and accuracy value of 71.73% in comparison with PPP alone derived 73.28% accuracy and 0.47 MCC (Table 5).

**Table 4:** Combined performance statistics of SVM employing solo features and hybrid approaches in predicting N-glycosites (using balanced dataset).

Feature	Sensitivity (%)	Specificity (%)	Accuracy (%)	MCC (%)	AUC (%)
CPP	59.81	64.49	62.15	0.24	0.65019
CPP+SS	63.55	69.16	66.36	0.33	0.68731
CPP+ASA	71.03	69.16	70.09	0.40	0.77203
CPP+SS+ASA	70.09	67.29	68.69	0.37	0.71159
**BPP**	**79.44**	**80.37**	**79.91**	**0.60**	**0.88322**
BPP+SS	82.24	80.37	81.31	0.63	0.88453
**BPP+ASA**	**84.11**	**81.31**	**82.71**	**0.65**	**0.89807**
BPP+SS+ASA	84.11	80.37	82.24	0.65	0.88497
PPP	76.42	69.81	73.11	0.46	0.76833
PPP+SS	75.70	71.03	73.36	0.47	0.78880
PPP+ASA	77.57	71.03	74.30	0.49	0.78636
PPP+SS+ASA	75.70	71.96	73.83	0.48	0.79334

Footnotes: BPP- Binary profile of patterns, CPP- Composition profile of patterns, PPP- PSSM profile of patterns, MCC- Matthews correlation coefficient, AUC- Area under curve, SS-secondary structure and ASA- Accessible surface area.

**Table 5:** Combined performance statistics of SVM classifiers employing solo features and hybrid approaches in predicting O-glycosites (using balanced dataset).

Feature	Sensitivity (%)	Specificity (%)	Accuracy (%)	MCC (%)	AUC (%)
CPP	68.10	72.41	70.26	0.41	0.74071
CPP+SS	70.69	71.55	71.12	0.42	0.75743
CPP+ASA	67.24	75.00	71.12	0.42	0.75780
CPP+SS+ASA	72.41	75.00	73.71	0.47	0.76955
BPP	66.38	67.24	66.81	0.34	0.73023
BPP+SS	69.83	68.10	68.97	0.38	0.74160
BPP+ASA	77.59	61.21	69.40	0.39	0.71143
BPP+SS+ASA	65.52	72.41	68.97	0.38	0.73766
**PPP**	**75.00**	**71.55**	**73.28**	**0.47**	**0.81250**
PPP+SS	73.28	73.28	73.28	0.47	0.76806
PPP+ASA	74.14	71.55	72.84	0.46	0.77341
**PPP+SS+ASA**	**77.59**	**69.83**	**73.71**	**0.48**	**0.76925**

Footnotes: BPP- Binary profile of patterns, CPP- Composition profile of patterns, PPP- PSSM profile of patterns, MCC- Matthews correlation coefficient, AUC- Area under curve, SS-secondary structure and ASA- Accessible surface area.

### Prediction Performance of SVM Using Realistic Datasets

For any machine learning technique, learning of datasets is very easy when both positive and negative instances are equal in number. Nevertheless, in case of glycoproteins, the negative instances could be much more than the positive instances in a protein sequence. Therefore, in order to judge accuracy and applicability of our SVM prediction schemes on realistic datasets of users, in parallel we have calculated the performances of aforementioned SVM models using realistic datasets. As was seen in case of models optimized with balanced datasets, BPP based SVM models performed better in case of realistic datasets and could achieve a maximum MCC 0.48 and 0.51 with accuracy value of 82.03% and 86.39% for prediction of N-glycosites using solo feature based and hybrid models (BPP+ASA), respectively. Similarly, O-glycosites could also be predicted with reasonably high accuracy of 70.24% and 89.69% (with corresponding maximum MCC values of 0.19 and 0.50) using CPP and CPP+ASA based models, respectively. Surprisingly, while using realistic datasets, predictions for O-glycosites were better with CPP based models in contrast to PPP based models that fared well in case of balanced datasets (Table 5, Table S1, Figure S4). Infact inclusion of surface accessibility features in combination with CPP in O-glycosites prediction scheme could enhance maximum MCC value of prediction by 2.5 fold (Table S1, Figure S4) indicating that surface accessibility alone could indeed be a useful criterion in glycosites prediction models discussed here. Further, the observed poorer performance of SVM with realistic datasets than with the balanced datasets of course is due to the inherent learning biases of realistic datasets. However, overall our SVM models optimized with realistic datasets fared reasonably well in predicting both N- and O- glycosites from realistic datasets (Table S1).

### Prediction Performance on Independent Datasets

Finally, the performance of best-optimized models as discussed before (Table 4, Table 5) were evaluated and compared with performances of NetOGlyc v 3.0, NetNGlyc, EnsembleGly against an independent set of experimentally verified prokaryotic glycoproteins (Table 6). Models developed and discussed in this study not only provided reasonably high accuracy (86.84% for N-glycosites with maximum MCC of 0.74 and 76.23% for O-glycosites with maximum MCC value of 0.53, respectively) but have convincingly outperformed performances of at least three of the well-known existing glycosites prediction tools as detailed in Table 6 in the context of prokaryotic glycosites prediction.

**Table 6:** Comparative performances of existing well-known glycosylation prediction tools and GlycoPP models on independent dataset of prokaryotic glycoproteins.

Prediction of N-glycosites						
Models (Threshold)	NetNglyc^1^ (0.5)	EnsembleGly^3^ (0.7)	GlycoPP-BPP (−0.1)	GlycoPP-CPP (0.3)	GlycoPP-PPP (−0.2)	GlycoPP-BPP+ASA
Sensitivity (%)	88.89	94.44	89.47	68.42	78.95	**89.47**
Specificity (%)	25.00	11.36	73.68	73.68	73.68	**84.21**
Accuracy (%)	43.55	35.48	81.58	71.05	76.32	**86.84**
MCC (%)	0.15	0.09	0.64	0.42	0.53	**0.74**
**Prediction of O-glycosites**						
**Models**	**NetOGlyc^2^ (0.1)**	**EnsembleGly^3^ (0.3)**	**GlycoPP-BPP (0.2)**	**GlycoPP-CPP (0.2)**	**GlycoPP-PPP** **(0)**	**GlycoPP-PPP+ASA**
Sensitivity (%)	100.00	6.67	72.13	72.55	77.05	**81.97**
Specificity (%)	3.19	93.05	73.77	68.18	70.49	**70.49**
Accuracy (%)	8.27	88.28	72.95	70.36	73.77	**76.23**
MCC (%)	0.04	**−**0.00	0.46	0.41	0.48	**0.53**

Footnotes: 1: http://www.cbs.dtu.dk/services/NetNGlyc/, 2: http://www.cbs.dtu.dk/services/NetOGlyc-3.0/, 3: http://turing.cs.iastate.edu/EnsembleGly/, BPP- Binary profile of patterns, CPP- Composition profile of patterns, PPP- PSSM profile of patterns, MCC- Matthews correlation coefficient, AUC- Area under curve, SS-secondary structure and ASA- Accessible surface area.

### Description of Web-server

The overall best performing models described in Table 6 are implemented in the form of a web-server GlycoPP available freely at http://www.imtech.res.in/raghava/glycopp/. The common gateway interface of GlycoPP is written using CGI/PERL script. This server allows for prediction of N- and O-glycosites in prokaryotic protein sequences. Predictions can be performed by the users at any of the user-defined thresholds ranging from −1.0 to 1.0 for optimizing SVM scores. Input is acceptable as single or multiple sequences in standard FASTA format.

## Discussion

In this study, we have developed new SVM based glycosites prediction models trained on and at least for N- and/or -O-glycosylated proteins belonging to six different archaeal and bacterial phyla namely, *Crenarchaeota*, *Euryarchaeota*, *Actinobacteria*, *Bacteroidetes*, *Firmicutes* and *Proteobacteria*. The overall best performing models are implemented at GlycoPP webserver available freely to the users (Figure 1). Our approach is similar to the existing models employed successfully for *in silico* identification of glycosites in eukaryotic glycoproteins [13], [14]. The webserver allows users to identify probable sites of N- and O-glycosylation in proteins belonging to or to the similar bacteria or archaea as described above, much more confidently than possible with the existing tools of similar nature. In this study, we observed that BPP models (containing single sequence information) were more efficient in discrimination of N-glycosylated and non-glycosylated sequences irrespective of their training on balanced or realistic datasets for the presence of a defined consensus-sequon NX(S/T) in all N-glycosites. Whereas, in case of O-glycosylation, multiple sequence information based PPP models performed better as the sole classifying feature. Possibly, for the lack of a defined consensus-sequon for most O-glycosites (except in phylum *Bacteroidetes*), [6], [24], PSSM derived profiles could well be more informative and useful for O-glycosites prediction. In our study, average surface accessibility emerged as a more useful criterion than secondary structure around glycosylated residues in most of our hybrid prediction approaches. The tool in its existing form would be useful for both single protein and proteome scale analysis. However, users are encouraged to supplement these results with other complementary evidences like presence of signal peptides, transmemebrane domains, sub-cellular localization of the proteins, presence of certain OSTs or GTs in the genome of the organism to indicate likely type and mode of glycosylation, known glycosylation in a close homologue and available experimental data on type of linkages, attached sugars etc., for best interpretation of the results obtained and also to decipher the biological significance of the same. The datasets used in this study are currently the largest and the most extensive available, yet inclusion of more validated sequences or features may further enhance the prediction accuracy, in future.

Further, the preliminary information gleaned from various organism-, phylum- and domain- specific weblogos of prokaryotic glycoproteins, suggest that sequence context of bacterial and archaeal N-glycosites not only differs from eukaryotic ones but they may vary between archaea and bacteria as well (Figures S1). In view of the understanding that the archaeal OST could be evolutionarily closer to eukaryotic OST [41], it may be beneficial to develop prediction tools separately for archaea and bacteria in future, when sufficient experimental data is available. Similarly, the approach could be extended to different phyla under domain Bacteria where novel sequons for N- and O-glycosites seem to be conserved with in a phylum. For example, preference for an acidic residue at -2 position in sequon for N-glycosylation among epsilonbacteria like *Campylobacter* and *Helicobacter* and novel O-glycosylation sequon D(S/T)(A/I/L/V/M/T) in phylum *Bacteroidetes* (Figures S2, S3) indicate that glycan and/or acceptor sequence specificities of OSTs/GTs could be conserved within a close group of bacteria and archaea. Therefore, in future it will be desirable to develop tools where prediction could be made taking in to account the glycan and/or acceptor sequence specificities of such individual protein glycosyltransferases of prokaryotes. However, as most of the OSTs involved in *en-bloc* N- and O- glycosylation both in archaea and bacteria including AglB, PglB, PglL and their homologues have been shown to have relaxed glycan specificity, the correlation between acceptor sequence specificity and glycan specificity of these enzymes may not be straight (Table 2), [37], [42], [43]. In this context, it could be speculated that in prokaryotes a complex inter-play of available biosynthesis machinery of certain precursor sugars, corresponding glycans, presence of certain OSTs/GTs along with their fine tuned specificities or subtle preferences towards given glycans and/or acceptor sequences may define protein glycosylation under given conditions.

### Supporting Information

The various datasets used in this study are available in downloadable format at http://www.imtech.res.in/raghava/glycopp/suppli.html.

## Supporting Information

Figure S1 Weblogos for archaeal N-glycosites (panel A) and bacterial N-glycosites (panel B).
(TIF)

Figure S2 Weblogos depicting two sequons for bacterial N-glycosites: (D/E)X_1_NX(S/T) in *Campylobacter* (panel A) and NX(S/T) in *Haemophilus* (panel B). Panel D represents typical eukaryotic mucin like sequence context around O-glycosites of mycobacterial glycoproteins whereas O-glycosites in *Campylobacter* is Ser, Gly rich as shown in panel C.
(TIF)

Figure S3 Conserved sequon D(S/T)A/I/L/V/M/T at O-glycosites in glycoproteins belonging to all major representatives: *Bacteroides* (panel A). *Flavobacterium* (panel B) and *Paedobacter* (panel C) of phylum *Bacteroidetes* (panel D).
(TIF)

Figure S4 ROC plots for various hybrid models for prediction of N-glycosites (panel A & B) and O-glycosites (panel C & D) using balanced datasets and realistic datasets, respectively. The Area Under Curve (AUC) depicts relative trade-offs between true positives and false positives.
(TIF)

Table S1 Combined prediction performance of SVM employing solo features and hybrid approaches (using realistic datasets).
(DOC)

## References

[1] Messner P (2004). Prokaryotic glycoproteins: unexplored but important.. Journal of Bacteriology.

[2] Abu-Qarn M, Eichler J, Sharon N (2008). Not just for Eukarya anymore: protein glycosylation in Bacteria and Archaea.. Curr Opin Struct Biol.

[3] Lechner J, Wieland F (1989). Structure and biosynthesis of prokaryotic glycoproteins.. Annu Rev Biochem.

[4] Upreti RK, Kumar M, Shankar V (2003). Bacterial glycoproteins: functions, biosynthesis and applications.. Proteomics.

[5] Varki A (1993). Biological roles of oligosaccharides: all of the theories are correct.. Glycobiology.

[6] Bhat AH, Mondal H, Chauhan JS, Raghava GP, Methi A (2012). ProGlycProt: a repository of experimentally characterized prokaryotic glycoproteins.. Nucleic Acids Res.

[7] Benz I, Schmidt MA (2002). Never say never again: protein glycosylation in pathogenic bacteria.. Molecular Microbiology.

[8] Dobos KM, Khoo KH, Swiderek KM, Brennan PJ, Belisle JT (1996). Definition of the full extent of glycosylation of the 45-kilodalton glycoprotein of Mycobacterium tuberculosis.. Journal of Bacteriology.

[9] Roy K, Hamilton D, Ostmann MM, Fleckenstein JM (2009). Vaccination with EtpA glycoprotein or flagellin protects against colonization with enterotoxigenic Escherichia coli in a murine model.. Vaccine.

[10] Jennings MP, Jen FE, Roddam LF, Apicella MA, Edwards JL (2011). Neisseria gonorrhoeae pilin glycan contributes to CR3 activation during challenge of primary cervical epithelial cells.. Cell Microbiol.

[11] Hansen JE, Lund O, Tolstrup N, Gooley AA, Williams KL (1998). NetOglyc: prediction of mucin type O-glycosylation sites based on sequence context and surface accessibility.. Glycoconj J.

[12] Julenius K, Molgaard A, Gupta R, Brunak S (2005). Prediction, conservation analysis, and structural characterization of mammalian mucin-type O-glycosylation sites.. Glycobiology.

[13] Gupta R, Brunak S (2002). Prediction of glycosylation across the human proteome and the correlation to protein function.. Pac Symp Biocomput.

[14] Caragea C, Sinapov J, Silvescu A, Dobbs D, Honavar V (2007). Glycosylation site prediction using ensembles of Support Vector Machine classifiers.. BMC Bioinformatics.

[15] Hamby SE, Hirst JD (2008). Prediction of glycosylation sites using random forests.. BMC Bioinformatics.

[16] Hanna ES, Roque-Barreira MC, Bernardes ES, Panunto-Castelo A, Sousa MV (2007). Evidence for glycosylation on a DNA-binding protein of *Salmonella enterica*.. Microb Cell Fact.

[17] Herrmann JL, Delahay R, Gallagher A, Robertson B, Young D (2000). Analysis of post-translational modification of mycobacterial proteins using a cassette expression system.. FEBS Lett.

[18] Balonova L, Hernychova L, Mann BF, Link M, Bilkova Z (2010). Multimethodological approach to identification of glycoproteins from the proteome of *Francisella tularensis*, an intracellular microorganism.. J Proteome Res.

[19] Ghoshal A, Mukhopadhyay S, Demine R, Forgber M, Jarmalavicius S (2009). Detection and characterization of a sialoglycosylated bacterial ABC-type phosphate transporter protein from patients with visceral leishmaniasis.. Glycoconj J.

[20] Dell A, Galadari A, Sastre F, Hitchen P (2010). Similarities and differences in the glycosylation mechanisms in prokaryotes and eukaryotes.. Int J Microbiol.

[21] Nothaft H, Szymanski CM (2010). Protein glycosylation in bacteria: sweeter than ever.. Nat Rev Microbiol.

[22] Marino K, Bones J, Kattla JJ, Rudd PM (2010). A systematic approach to protein glycosylation analysis: a path through the maze.. Nat Chem Biol.

[23] Kowarik M, Young NM, Numao S, Schulz BL, Hug I (2006). Definition of the bacterial N-glycosylation site consensus sequence.. Embo Journal.

[24] Fletcher CM, Coyne MJ, Comstock LE (2011). Theoretical and experimental characterization of the scope of protein O-glycosylation in Bacteroides fragilis.. Journal of Biological Chemistry.

[25] Abu-Qarn M, Eichler J (2007). An analysis of amino acid sequences surrounding archaeal glycoprotein sequons.. Archaea.

[26] Ben-Dor S, Esterman N, Rubin E, Sharon N (2004). Biases and complex patterns in the residues flanking protein N-glycosylation sites.. Glycobiology.

[27] Petrescu AJ, Milac AL, Petrescu SM, Dwek RA, Wormald MR (2004). Statistical analysis of the protein environment of N-glycosylation sites: implications for occupancy, structure, and folding.. Glycobiology.

[28] Kowarik M, Numao S, Feldman MF, Schulz BL, Callewaert N (2006). N-linked glycosylation of folded proteins by the bacterial oligosaccharyltransferase.. Science.

[29] Chauhan JS, Mishra NK, Raghava GP (2010). Prediction of GTP interacting residues, dipeptides and tripeptides in a protein from its evolutionary information.. BMC Bioinformatics.

[30] Agarwal S, Mishra NK, Singh H, Raghava GP (2011). Identification of mannose interacting residues using local composition.. PLoS One.

[31] Kumar M, Gromiha MM, Raghava GP (2008). Prediction of RNA binding sites in a protein using SVM and PSSM profile.. Proteins.

[32] McGuffin LJ, Bryson K, Jones DT (2000). The PSIPRED protein structure prediction server.. Bioinformatics.

[33] Garg A, Kaur H, Raghava GP (2005). Real value prediction of solvent accessibility in proteins using multiple sequence alignment and secondary structure.. Proteins.

[34] Joachims T (1999). Making large-Scale SVM Learning Practical In: Advances in Kernel Models - Support Vector Learning, B. Schölkopf and C. Burges and A. Smola (ed.), MIT-Press..

[35] Kalita MK, Nandal UK, Pattnaik A, Sivalingam A, Ramasamy G (2008). CyclinPred: a SVM-based method for predicting cyclin protein sequences.. PLoS One.

[36] Ansari HR, Raghava GP (2010). Identification of conformational B-cell Epitopes in an antigen from its primary sequence.. Immunome Res.

[37] Ielmini MV, Feldman MF (2011). Desulfovibrio desulfuricans PglB homolog possesses oligosaccharyltransferase activity with relaxed glycan specificity and distinct protein acceptor sequence requirements.. Glycobiology.

[38] Choi KJ, Grass S, Paek S, St Geme JW, 3rd, Yeo HJ (2010). The Actinobacillus pleuropneumoniae HMW1C-like glycosyltransferase mediates N-linked glycosylation of the Haemophilus influenzae HMW1 adhesin.. PLoS One.

[39] Jervis AJ, Langdon R, Hitchen P, Lawson AJ, Wood A (2010). Characterization of N-linked protein glycosylation in Helicobacter pullorum.. Journal of Bacteriology.

[40] Rangarajan ES, Bhatia S, Watson DC, Munger C, Cygler M (2007). Structural context for protein N-glycosylation in bacteria: The structure of PEB3, an adhesin from Campylobacter jejuni.. Protein Sci.

[41] Maita N, Nyirenda J, Igura M, Kamishikiryo J, Kohda D (2010). Comparative structural biology of eubacterial and archaeal oligosaccharyltransferases.. Journal of Biological Chemistry.

[42] Faridmoayer A, Fentabil MA, Haurat MF, Yi W, Woodward R (2008). Extreme substrate promiscuity of the *Neisseria* oligosaccharyl transferase involved in protein O-glycosylation.. J Biol Chem.

[43] Calo D, Kaminski L, Eichler J (2010). Protein glycosylation in Archaea: sweet and extreme.. Glycobiology.

