# Supplementary figures and images for: GlycoPP: A Webserver for Prediction of N- and O-Glycosites in Prokaryotic Protein Sequences
Source: PLoS One. 2012 Jul 9;7(7):e40155. doi: 10.1371/journal.pone.0040155 (PMC3392279; doi:10.1371/journal.pone.0040155)

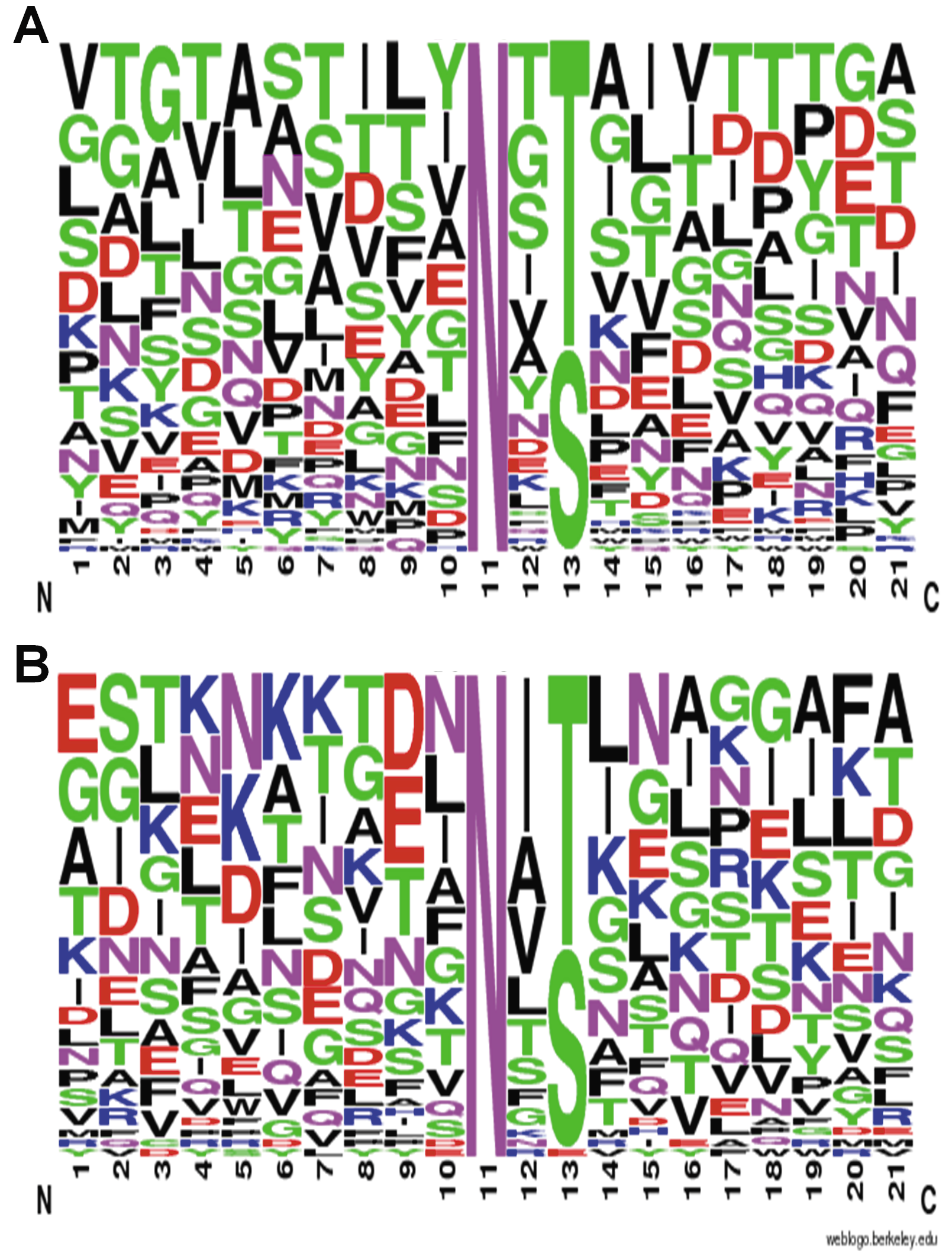

Supplement: Figure S1 — Weblogos for archaeal N-glycosites (panel A) and bacterial N-glycosites (panel B). (TIF) [file pone.0040155.s001.tif]

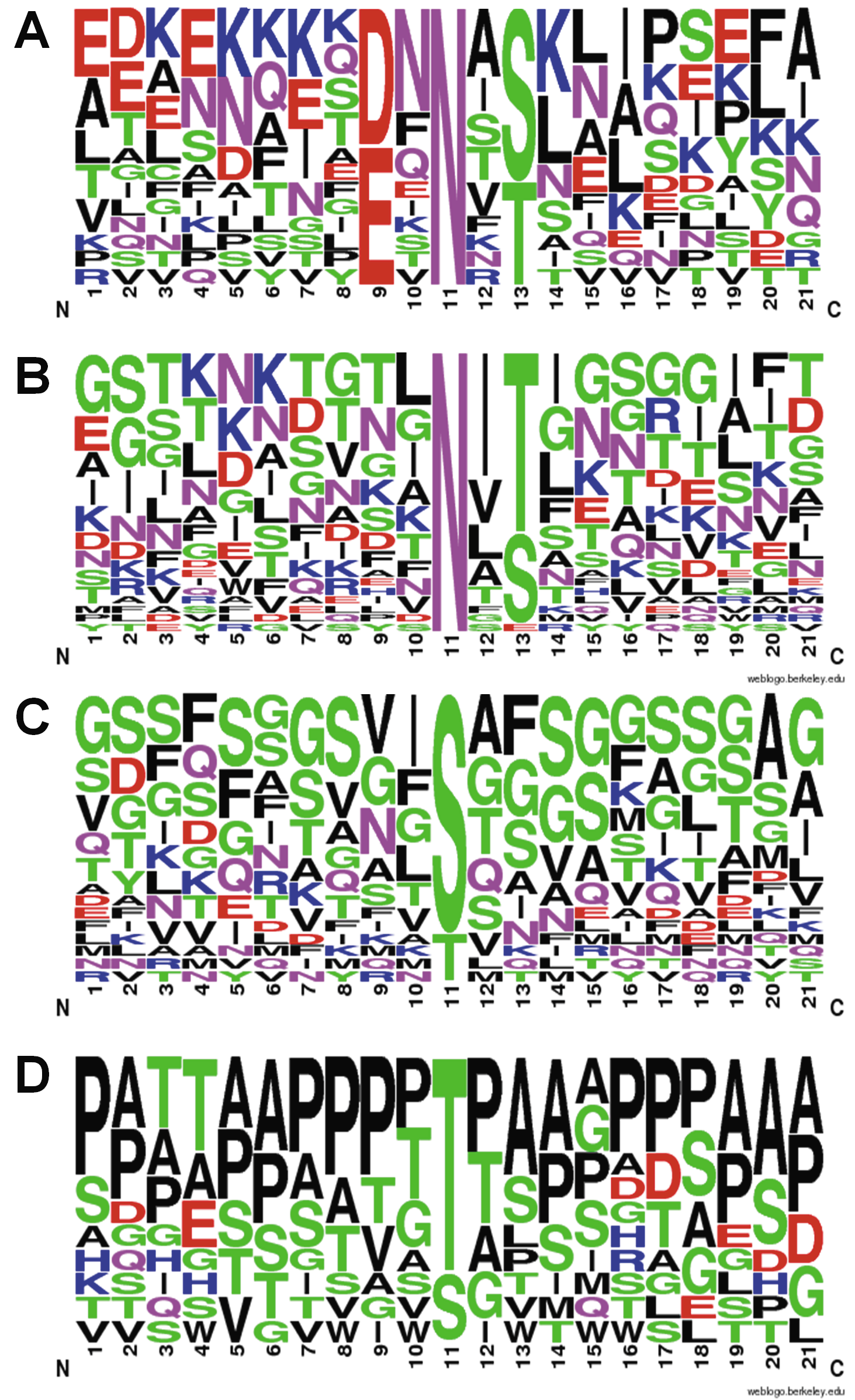

Supplement: Figure S2 — Weblogos depicting two sequons for bacterial N-glycosites: (D/E)X1NX(S/T) in Campylobacter (panel A) and NX(S/T) in Haemophilus (panel B). Panel D represents typical eukaryotic mucin like sequence context around O-glycosites of mycobacterial glycoproteins whereas O-glycosites in Campylobacter is Ser, Gly rich as shown in panel C. (TIF) [file pone.0040155.s002.tif]

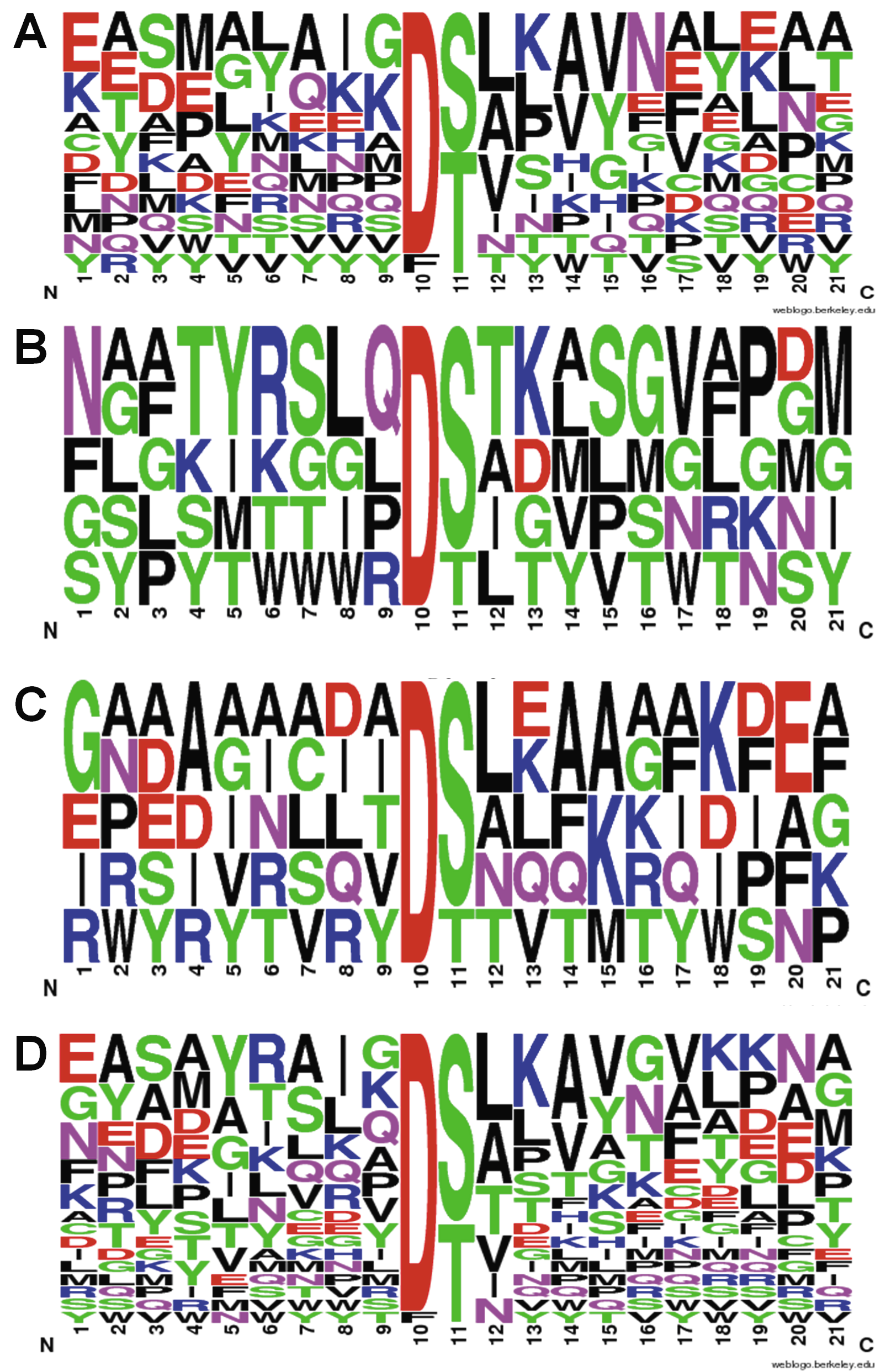

Supplement: Figure S3 — Conserved sequon D(S/T)A/I/L/V/M/T at O-glycosites in glycoproteins belonging to all major representatives: Bacteroides (panel A). Flavobacterium (panel B) and Paedobacter (panel C) of phylum Bacteroidetes (panel D). (TIF) [file pone.0040155.s003.tif]

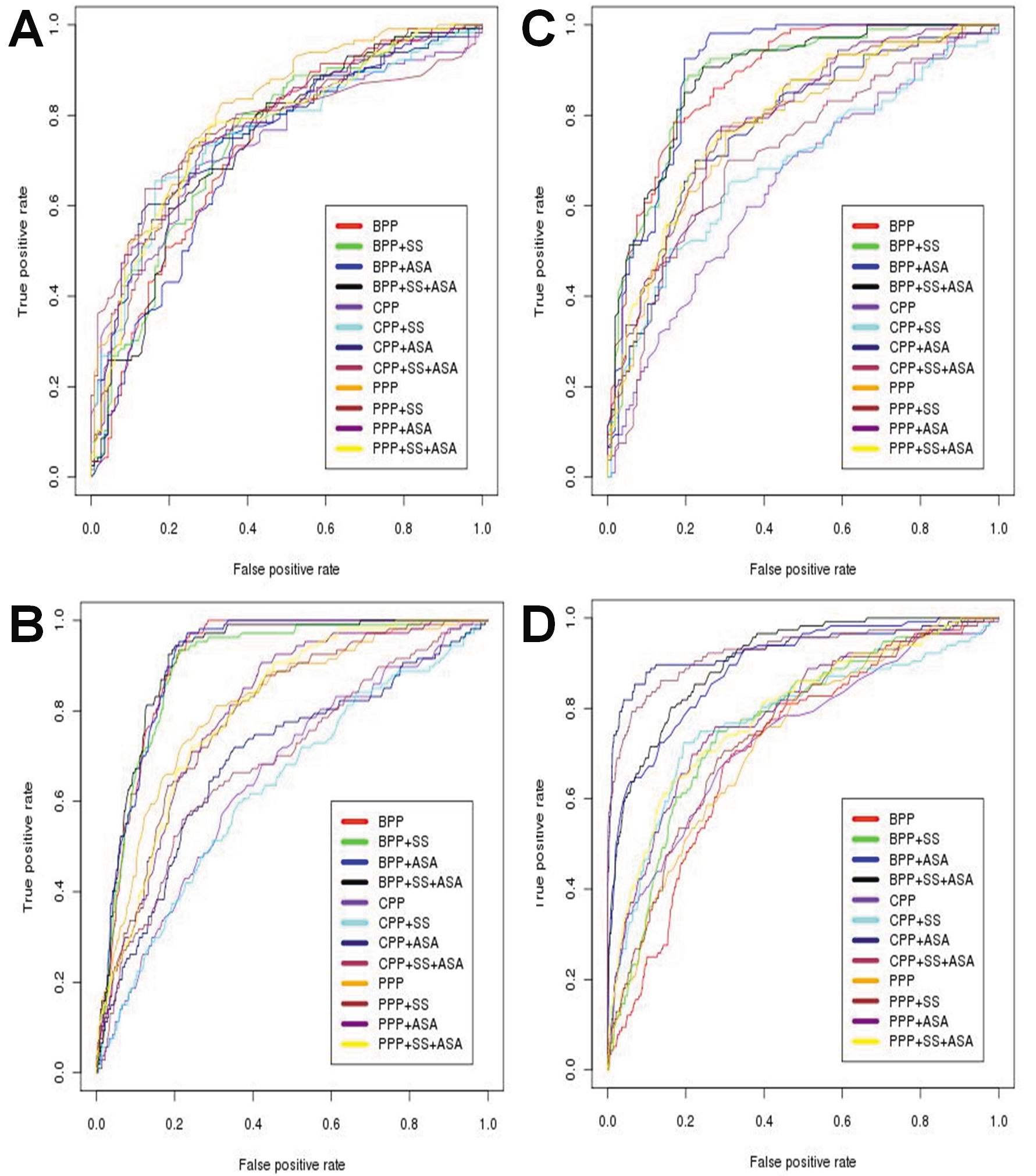

Supplement: Figure S4 — ROC plots for various hybrid models for prediction of N-glycosites (panel A & B) and O-glycosites (panel C & D) using balanced datasets and realistic datasets, respectively. The Area Under Curve (AUC) depicts relative trade-offs between true positives and false positives. (TIF) [file pone.0040155.s004.tif]
