# Supplementary material for: GlycoPP: A Webserver for Prediction of N- and O-Glycosites in Prokaryotic Protein Sequences
Source: PLoS One. 2012 Jul 9;7(7):e40155. doi: 10.1371/journal.pone.0040155 (PMC3392279; doi:10.1371/journal.pone.0040155)
Supplement: Table S1 — Combined prediction performance of SVM employing solo features and hybrid approaches (using realistic datasets). (DOC) [file pone.0040155.s005.doc]

**Table S1. Combined prediction performance of SVM employing solo features and hybrid approaches (using realistic datasets)**

| Type of  Glycosylation | SVM Input  feature | Sensitivity (%) | Specificity (%) | Accuracy (%) | MCC (%) | AUC (%) |
| --- | --- | --- | --- | --- | --- | --- |
| N-linked | BPP | 85.98 | 82.71 | 83.03 | 0.48 | 0.90 |
|  | BPP+SS | 85.05 | 82.41 | 82.67 | 0.47 | 0.89 |
|  | BPP+ASA | 89.72 | 81.21 | 82.03 | 0.48 | 0.91 |
|  | BPP+SS+ASA | 81.31 | 86.93 | 86.39 | 0.51 | 0.91 |
|  | CPP | 61.68 | 64.42 | 64.16 | 0.16 | 0.65 |
|  | CPP+SS | 59.81 | 63.32 | 62.98 | 0.14 | 0.63 |
|  | CPP+ASA | 69.16 | 66.93 | 67.15 | 0.22 | 0.69 |
|  | CPP+SS+ASA | 63.55 | 68.14 | 67.70 | 0.20 | 0.68 |
|  | PPP | 70.75 | 78.67 | 77.91 | 0.33 | 0.80 |
|  | PPP+SS | 66.36 | 77.59 | 76.50 | 0.29 | 0.79 |
|  | PPP+ASA | 71.03 | 75.68 | 75.23 | 0.31 | 0.79 |
|  | PPP+SS+ASA | 71.03 | 75.78 | 75.32 | 0.31 | 0.80 |
| O-linked | BPP | 68.10 | 68.68 | 68.65 | 0.18 | 0.71 |
|  | BPP+SS | 70.69 | 74.48 | 74.27 | 0.23 | 0.76 |
|  | BPP+ASA | 78.45 | 81.07 | 80.93 | 0.32 | 0.89 |
|  | BPP+SS+ASA | 80.17 | 83.65 | 83.46 | 0.36 | 0.90 |
|  | **CPP** | **68.10** | **70.37** | **70.24** | **0.19** | 0.74 |
|  | CPP+SS | 72.41 | 79.93 | 79.52 | 0.28 | 0.77 |
|  | CPP+ASA | 87.93 | 89.79 | 89.69 | 0.50 | 0.93 |
|  | CPP+SS+ASA | 83.62 | 87.91 | 87.68 | 0.44 | 0.92 |
|  | PPP | 62.93 | 69.82 | 69.45 | 0.16 | 0.73 |
|  | PPP+SS | 68.97 | 71.66 | 71.51 | 0.20 | 0.74 |
|  | PPP+ASA | 72.41 | 75.22 | 75.07 | 0.24 | 0.79 |
|  | PPP+SS+ASA | 70.69 | 75.52 | 75.26 | 0.24 | 0.79 |

**Footnotes: BPP- Binary profile of patterns, CPP- Composition profile of patterns, PPP- PSSM profile of patterns, MCC- Matthews correlation coefficient, AUC- Area under curve, SS-secondary structure and ASA- Accessible surface area**
